# Epithelial C5aR1 Signaling Enhances Uropathogenic *Escherichia coli* Adhesion to Human Renal Tubular Epithelial Cells

**DOI:** 10.3389/fimmu.2018.00949

**Published:** 2018-05-01

**Authors:** Yun Song, Kun-Yi Wu, Weiju Wu, Zhao-Yang Duan, Ya-Feng Gao, Liang-Dong Zhang, Tie Chong, Malgorzata A. Garstka, Wuding Zhou, Ke Li

**Affiliations:** ^1^Core Research Laboratory, The Second Affiliated Hospital, School of Medicine, Xi’an Jiaotong University, Xi’an, China; ^2^Medical Research Council (MRC) Centre for Transplantation, King’s College London, Guy’s Hospital, London, United Kingdom; ^3^Department of Nephrology, The Second Affiliated Hospital, School of Medicine, Xi’an Jiaotong University, Xi’an, China; ^4^Department of Urology, The Second Affiliated Hospital, School of Medicine, Xi’an Jiaotong University, Xi’an, China

**Keywords:** C5aR1, uropathogenic *Escherichia coli*, renal tubular epithelial cell, bacterial adhesion/invasion, mannosyl residues

## Abstract

Recent work in a murine model of ascending urinary tract infection has suggested that C5a/C5aR1 interactions play a pathogenic role in the development of renal infection through enhancement of bacterial adhesion/colonization to renal tubular epithelial cells (RTECs). In the present study, we extended these observations to human. We show that renal tubular epithelial C5aR1 signaling is involved in promoting uropathogenic *Escherichia coli* (UPEC) adhesion/invasion of host cells. Stimulation of primary cultures of RTEC with C5a resulted in significant increases in UPEC adhesion/invasion of the RTEC. This was associated with enhanced expression of terminal α-mannosyl residues (Man) (a ligand for type 1 fimbriae of *E. coli*) in the RTEC following C5a stimulation. Mechanism studies revealed that C5aR1-mediated activation of ERK1/2/NF-κB and upregulation of proinflammatory cytokine production (i.e., TNF-α) is at least partly responsible for the upregulation of Man expression and bacterial adhesion. Clinical sample studies showed that C5aR1 and Man were clearly detected in the renal tubular epithelium of normal human kidney biopsies, and UPEC bound to the epithelium in a d-mannose-dependent manner. Additionally, C5a levels were significantly increased in urine of urinary tract infection patients compared with healthy controls. Our data therefore demonstrate that, in agreement with observations in mice, human renal tubular epithelial C5aR1 signaling can upregulate Man expression in RTEC, which enhances UPEC adhesion to and invasion of RTEC. It also suggests the *in vivo* relevance of upregulation of Man expression in renal tubular epithelium by C5a/C5aR1 interactions and its potential impact on renal infection.

## Introduction

Urinary tract infections (UTIs) remain among the most common infectious diseases worldwide (1). Globally, there have been estimated over 250 million cases of UTI each year (2). It is frequent in women and children and causes a particular problem for patients with diabetes and renal transplants as well as catheterized patients (3–5). The most common causative organism is uropathogenic *Escherichia coli* (UPEC), which is responsible for around 80% of all cases. UPEC express a variety of fimbrial adhesins (e.g., type 1, P, and Dr fimbriae) that enable them to bind to glycoproteins or glycolipids on urinary tract epithelial cells (the critical initial step in early colonization). Upon contact with the epithelial cells, UPEC liberate toxins (e.g., α-hemolysin and cytotoxic necrotizing factor-1) which mediate direct injury to the cells, disrupting the epithelial barrier, and opening access to the underlying tissue. In addition, UPEC can be uptaken by the epithelial cells (6–8), which could evade host defenses and establish reservoir that may act as a potential source for recurrent infections (9).

Recent research has suggested that the epithelial cell lining of urinary tract is not simply a passive target for infection, oxidative stress, and toxical drug, but may actively participate in the innate immune response (10). For example, in response to the invasion of pathogen or other injuries, tubular epithelial cells produce a number of proinflammatory mediators, which can activate and recruit immune cells to the site of infection and injury, contributing to innate immunity; however, excessive inflammatory responses can also cause renal tubule cell injury (10–12). Human renal tubular epithelial cells (RTECs) produce various components of the complement system (e.g., C3, fB, and fH) (13–15), local production, and activation of complement have been implicated in the pathogenesis of many renal tubular disorders (16, 17).

Complement is an important element in innate immunity against pathogens, mainly through direct killing, opsonization of pathogen, and induction of local inflammation, which are mediated by C5b-9, C3b, and C5a, respectively. However, most UPEC strains are resistant to complement-mediated killing (18). Previous studies by us and others have suggested that complement activation in UTI has harmful effects on the host (7, 8, 19). It has been shown that depletion of complement in non-human primate decreases the degree of tissue damage during renal infection (19) and mice deficient in C3, a pivotal component of the complement cascade, are protected from the development of renal infection (7). C3b-opsonized *E. coli* enhances the invasion of human tubular epithelial cells through interaction with a complement regulatory protein CD46 expressed on the epithelial cell membrane (8).

Further studies in murine models of acute and chronic pyelonephritis have found that C5aR1 is required for the development of renal infection (20, 21). It has been shown that kidney infection is significantly reduced in mice with genetic deletion or through pharmacologic inhibition of C5aR1 following bladder inoculation with UPEC. This is associated with reduced expression of terminal α-mannosyl residues (Man; a ligand for type 1 fimbriae of *E. coli*) on the luminal surface of renal tubular epithelium and reduction of early UPEC colonization in these mice. These studies strongly suggest that C5a/C5aR1 interactions are an important pathogenic mechanism of ascending UTI. However, the relevance of these findings to human remains unknown.

In the present study, we extended our observations made in murine models to human. We investigated whether renal tubular epithelial C5aR1 signaling has impact on UPEC adhesion to and invasion of RTEC using primary cultures of human RTEC. We also examined the potential mechanisms by which epithelial C5aR1 signaling influences bacterial adhesion. Furthermore, we performed several *ex vivo* experiments using human kidney biopsies and urine samples to assess the *in vivo* relevance of *in vitro* observations. Our data demonstrate that epithelial C5aR1 signaling promotes UPEC adhesion to and invasion of human RTEC through upregulation of MR expression in the RTEC. It also suggests the *in vivo* relevance of upregulation of Man expression in renal tubular epithelium by C5a/C5aR1 interactions and its potential impact on renal infection.

## Materials and Methods

### Materials

We used the following reagents and materials: mouse anti-human C5aR1 (P12/1, for immunochemistry) (AbD Serotec, Oxford, UK), PE-conjugated anti-human C5aR1 (S5/1, for flow cytometric analysis) (Biolegend, San Diego, CA, USA); polyclonal rabbit anti-human ZO-1 and 4’,6-diamidino-2-phenylindole (DAPI) (Life Technologies Ltd., Beijing, China); goat anti-mouse alexa Fluor^®^ 488 (Jackson ImmunoResearch Lab Inc., West Grove, PA, USA); fluorescein-labeled *Galanthus nivalis* lectin (GNL) (Vector Laboratories, Peterborough, UK); cytopainter F-actin staining kit (Abcam, Cambridge, UK). BD™ cytometric bead array human inflammatory cytokines kit (to measure TNF-α, IL-8, IL-1β, and IL-6) and human C5a ELISA kit II (BD OptEIA™) (BD Biosciences, San Jose, USA). Antibody reagents used in signaling pathway studies [i.e., anti-phospho-ERK1/2 (Thr202/Tyr204), -IκBα (Ser32), -SAPK/JNK (Thr183/Tyr185), -Akt (Ser473), and anti-ERK1/2, -IκBα, -JNK, and -Akt antibodies] and ERK1/2 inhibitor U0126 were purchased from Cell Signaling Technology (Danvers, MA, USA). Cell culture medium, fetal calf serum (FCS), insulin-transferrin-selenium solution, gentamicin (Life Technologies Ltd.); recombinant human TNF-α (Peprotech, Suzhou, China); d-(+)-Mannose, hydrocortisone, tri-iodothyronine and tetramethylrhodamine (TRITC), protease inhibitor cocktail, LPS (from *E. coli* with serotype O55:B5, contains O antigen) (Sigma-Aldrich, Shanghai, China); collagenase type II (Worthington Bio. Co., USA); human recombinant C5a (with an endotoxin level of ≤0.1 EU per 1 µg of protein) (R&D systems). C5aR1 peptide antagonist [PMX53, Ac-Phe-cyclo (Orn-Pro-dCha-Trp-Arg)] and control peptide (random sequence) (synthesized by GenScript, Shanghai, China).

### Bacterial Strains

Uropathogenic *E. coli* strain J96 (serotype O4; K6), isolated from a human pyelonephritis patient was kindly provided by Dr. R. Welch, University of Wisconsin, USA. This strain expresses type 1 and P fimbriae and secrets α-hemolysins and the cytotoxic necrotizing factor-1 (18).

### Human Kidney Specimens and Ethics

Normal human kidney specimens were prepared from the unaffected pole of nephrectomized kidneys of patients who had renal tumors (*n* = 4). Each patient gave an informed consent about the present study in accordance with the Declaration of Helsinki. The protocol was approved by the Hospital Research Ethics Committee.

### Cell Cultures and Treatment

Human RTEC were isolated according to a previously described method (14). Briefly, renal cortical tissue obtained from normal pole of tumor nephrectomy specimens was cut into small fragments and digested with collagenase (type II, 750 U/mL) at 37°C for 15 min and passed through a series of mesh sieves. Tubular cells were isolated by centrifugation and grown in DMEM/F12 with 5% FCS supplemented with insulin (5 µg/mL), transferrin (5 µg/mL), selenium (5 ng/mL), hydrocortisone (40 ng/mL), and tri-iodothyronine (10^−12^ M). The cultured cells exhibited cuboidal morphology (by phase contrast microscopy) and were positive for the expression of alkaline phosphatase and cytokeratin (Figure S1 in Supplementary Material). Experiments were performed with cells up to the sixth passage. Results were obtained from RTEC cultured from the kidney of three different donors. For experiments, confluent layers of RTEC were incubated with C5a (0–50 nM), LPS (800 ng/mL), TNF-α (10 ng/mL), or C5aR1 peptide antagonist (PMX53, 5 µM) for 24 h at 37°C.

### Assessment of Bacterial Binding and Internalization

Bacterial plate count assay: The assay was performed as described previously (8). Briefly, RTEC grown in 24-well plates were pre-incubated with or without C5a or other stimuli for 24 h and then incubated with bacteria [J96, 2 × 10^6^ colony forming units (c.f.u.)/well] for an additional 1 h at 37°C. For bacterial binding, cells were vigorously washed to remove unattached bacteria and lysed with sterile H_2_O. For bacterial internalization, after incubation with bacteria for 1 h, RTEC were washed three times and then incubated in culture medium containing 100 µg/mL gentamicin for 1 h to kill extra-cellular bacteria. Cells were then washed and lysed with sterile H_2_O, the lysate was plated onto cysteine lactose electrolyte deficient (CLED) plates (Oxoid). The agar plates were incubated at 37°C for overnight and the colonies were manually counted. RTEC protein concentrations were measured using the Coomassie (Bradford) protein assay kit according to the manufacturer’s instructions. Results were expressed as colony c.f.u./mg of cell protein. In each experiment, assays were performed in quadruplicate.

Fluorescence microscopy analysis: RTEC monolayers grown on coverslips in 24-well plates were incubated with TRITC-conjugated J96 (2 × 10^6^ c.f.u./well) for 1 h at 37°C. Cells were vigorously washed to remove unattached bacteria and stained with DAPI, then viewed and imaged with the Leica SP8 system. Bound bacteria and RTEC numbers were counted at ×200 magnification, and results were expressed as number of bacteria per 10^3^ tubule cells. To confirm the intracellular location of the bacteria, after incubation with labeled bacteria, the monolayers were washed then fixed in 4% formaldehyde and permeabilized with 0.1% TritonX-100 before incubating with green fluorescence -conjugated F-actin (Abcam) for 30 min. Confocal microscopy was carried out using cross-sectional analysis. To demonstrate the co-localization of bacteria with Man, after incubation with labeled bacteria, cells were vigorously washed to remove unattached bacteria and stained with DAPI and fluorescein-labeled GNL, then viewed and imaged with the Leica SP8 system. Two to three viewing fields, randomly selected from each coverslip at ×630 magnification, were examined.

### Assessment of Bacterial Migration in RTEC

Renal tubular epithelial cell were cultured on 12-well transwell filters (with a 3 µm transparent membrane insert) (Corning) and allowed to grow to confluent monolayers for 10 days. The tightness of the cell layers was verified by addition of 500 µL culture medium in the upper compartment followed by incubation at 37°C for 6 h. No medium was found in the lower compartment when cells confluent. Cells were stimulated with or without C5a (10 nM) for 24 h and then incubated with J96 (2 × 10^6^ c.f.u./well) for up to 3 h at 37°C. Bacterial migration was measured by plating out the culture medium collected from lower chamber on CLED plate and counting the colonies formed on the plate the next day. RTEC membrane integrity was assessed by immunochemistry and western blot analyses for tight junction marker ZO-1.

### Assessment of Man Expression in Cultured RTEC

Man expression in cultured RTEC was assessed by a fluorescence intensity-based microplate assay or fluorescence microscopic analysis as we described previously (20). For the microplate assay, RTEC grown in 24-well plates were incubated with or without C5a and/or LPS for 24 h at 37°C, then with fluorescein-labeled GNL (20 µg/mL in PBS) for 1 h at 37°C. Cells were then vigorously washed and lysed with sterile H_2_O. The fluorescence in the lysate was measured using a fluorescence plate reader (SpectraMax i3, Molecular Devices) with an excitation wavelength of 490 nm and results expressed as relative fluorescence units. Fluorescence microscopic analysis was used to detect surface Man on RTEC. RTEC grown on the coverslips were pre-treated with C5a and/or LPS and stained with fluorescein-labeled GNL and DAPI. Images were taken by the confocal microscope (Leica SP8) under ×100 magnification. The percentage of positive staining area in each image was calculated by using Image J software (Image J 1.41). Three to four viewing fields randomly selected from each coverslip were examined.

### α-Mannosidase Treatment

Renal tubular epithelial cell were pre-incubated with FCS-free medium for 2 h and then incubated with α-mannosidase or control enzyme (β-galactosidase) (5 mM) in glycol buffer at 37°C for 1 h. FCS-containing culture medium was added to neutralize the effect of mannosidase (22). Cells were then subjected to measuring changes in Man expression and bacterial binding.

### Western Blot

Human RTECs were primarily cultured on six-well plates and grown to confluence. Cells were incubated with C5a and lysed at indicated times. Equal amounts of protein (40 µg per lane) were subjected to SDS-PAGE electrophoresis, and transferred onto PVDF membranes. The membranes were incubated with primary antibody at 4°C overnight and followed by incubation with HRP-conjugated secondary antibody. Protein bands were visualized by Amersham ECL Select™ detection reagent (GE Healthcare Life Sciences, USA).

### Detection of C5aR1 Expression in Kidney Tissue by Immunohistochemistry

Paraffin-embedded human kidney tissue sections (4 µm thick, from four different donors) were deparaffinized, rehydrated, and subjected to microwave-based antigen retrieval in citrate buffer. Endogenous peroxidases were blocked with 0.3% (v/v) H_2_O_2_ in PBS for 10 min. Non-specific immunoglobulin binding sites were blocked with normal goat serum. Sections were subsequently incubated with mouse anti-human C5aR1 primary antibody (P12/1, AbD Serotec) (1:300 dilution) at 4°C overnight. Non-specific mouse IgG2a served as a negative control. Sections were then incubated for 1 h with HRP-conjugated goat anti-mouse secondary antibody (Jackson ImmunoResearch Lab Inc., 1:1,000 dilution) at room temperature. The antibody staining was visualized by DAB solution according to manufacturer’s instructions. The stained sections were imaged with automatic slide scanner (Axio Scan Z1, Zeiss, Germany).

### UPEC *In Situ* Binding to Kidney Tissue

Frozen sections (4 µm) of OCT-embedded human kidney samples (*n* = 4) were immobilized on Superfrost™ Plus microscope slides (Fisher Scientific) and were rehydrated in PBS for 5 min and incubated with PBS containing 0.1% BSA for 1 h, and then with TRITC-labeled J96 (2 × 10^7^ c.f.u./mL, pre-incubated with 1 or 5% Glucose or d-mannose for 30 min and resuspended in PBS containing 0.1% BSA) for 1 h at 37°C. Sections were then gently washed five times to remove unattached bacteria and stained with DAPI and fluorescein-labeled GNL, then viewed and imaged with the Leica SP8 system. Bacterial colonies were manually counted at ×200 magnification and results were expressed as number of colonies per field. Five viewing fields at cortex area for each kidney section were examined.

### Statistical Analysis

Data are shown as mean ± SEM. Mann–Whitney test or Unpaired Student’s *t*-test was used to compare the means of two groups. One-way or two-way ANOVA was used to compare the means of more than two independent groups. All the analyses were performed using Graphpad Prism 7 software. *P* < 0.05 was considered to be significant.

## Results

### C5a Enhances UPEC Adhesion and Invasion of RTEC by Stimulating the Epithelial Cells

We first examined the expression of C5aR1 in primary cultures of human RTEC by performing immunochemical staining, flow cytometry, and RT-PCR. C5aR1 expression was clearly detected in the RTEC by the three methods, further confirming that C5aR1 is expressed in human RTEC (Figures S2A–C in Supplementary Material). We also examined whether C5R2 (an additional receptor for C5a) is expressed in the RTEC by RT-PCR, which showed a negative result (Figure 2C in Supplementary Material). Therefore, any effects of C5a stimulation in our RTEC preparations likely through interaction with C5aR1.

**Figure 1:**
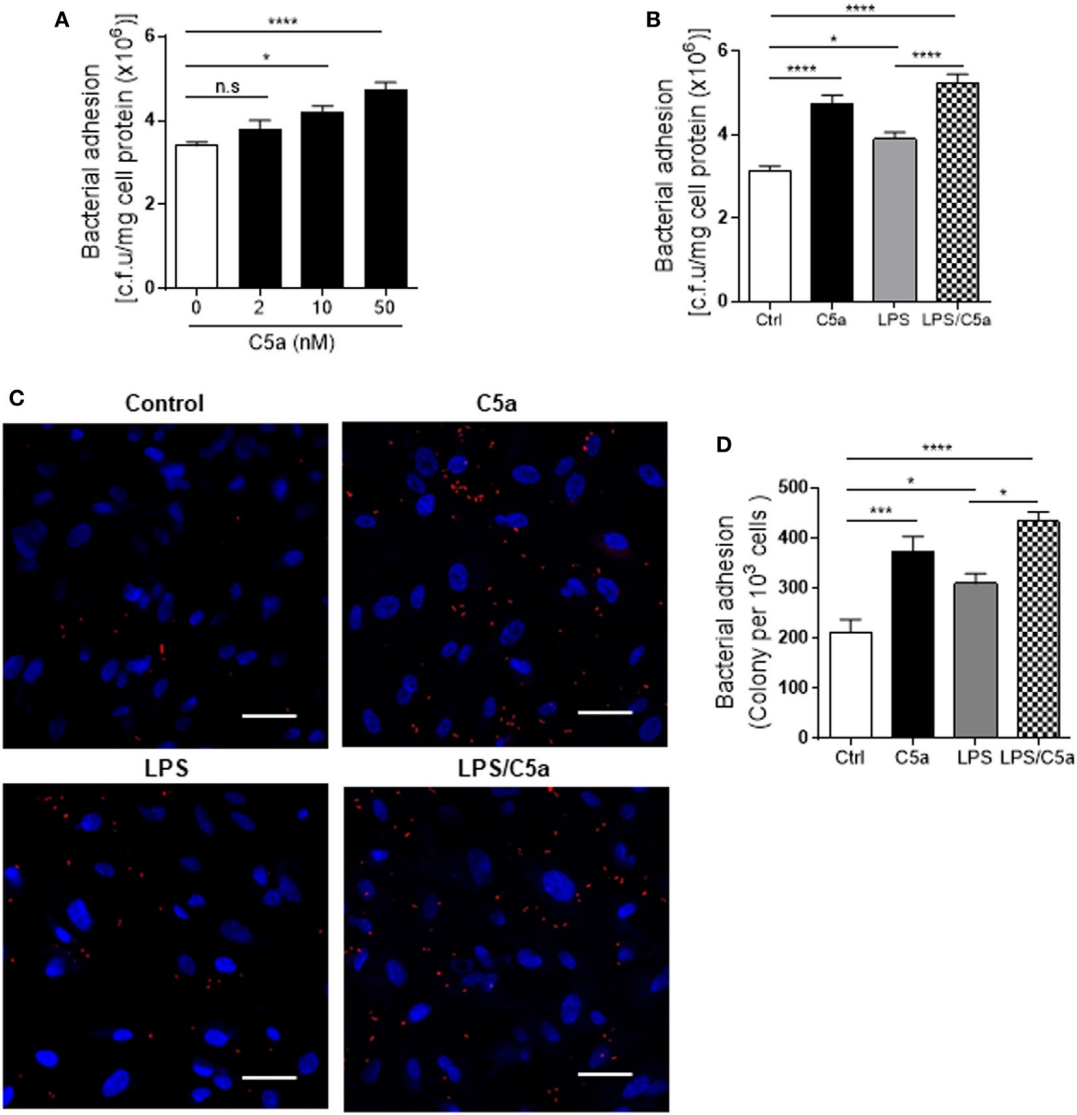
Effect of C5a stimulating renal tubular epithelial cell (RTEC) on bacterial adhesion to the RTEC. RTEC were pre-treated with C5a (as indicated or otherwise 10 nM) and/or LPS (800 ng/mL) for 24 h and subjected to assessment of bacteria binding to RTEC. **(A,B)** Bacterial adhesion to RTEC, evaluated by bacterial plate count assay. **(C,D)** Bacterial adhesion to RTEC, evaluated by fluorescence microscopy analysis. **(C)** Representative fluorescence images of tri-iodothyronine and tetramethylrhodamine-labeled J96 adhesion to RTEC, J96 (red), and 4’,6-diamidino-2-phenylindole (blue). Scale bars, 50 µm. **(D)** Quantification of bacteria adhesion to RTEC corresponding to the images in **(C)**. Results were expressed as number of bacteria per 10^3^ RTEC. **(A,B,D)** data were analyzed by one-way ANOVA with Tukey’s multiple comparisons test [**(A)** *n* = 12 individual wells per group, **(B)** *n* = 8 individual wells per group, **(C)** *n* = 6 individual images (×200 magnification) from two coverslips per group]. All results shown are representative of three independent experiments. *P < 0.05, ***P < 0.001, ****P < 0.0001.

**Figure 2:**
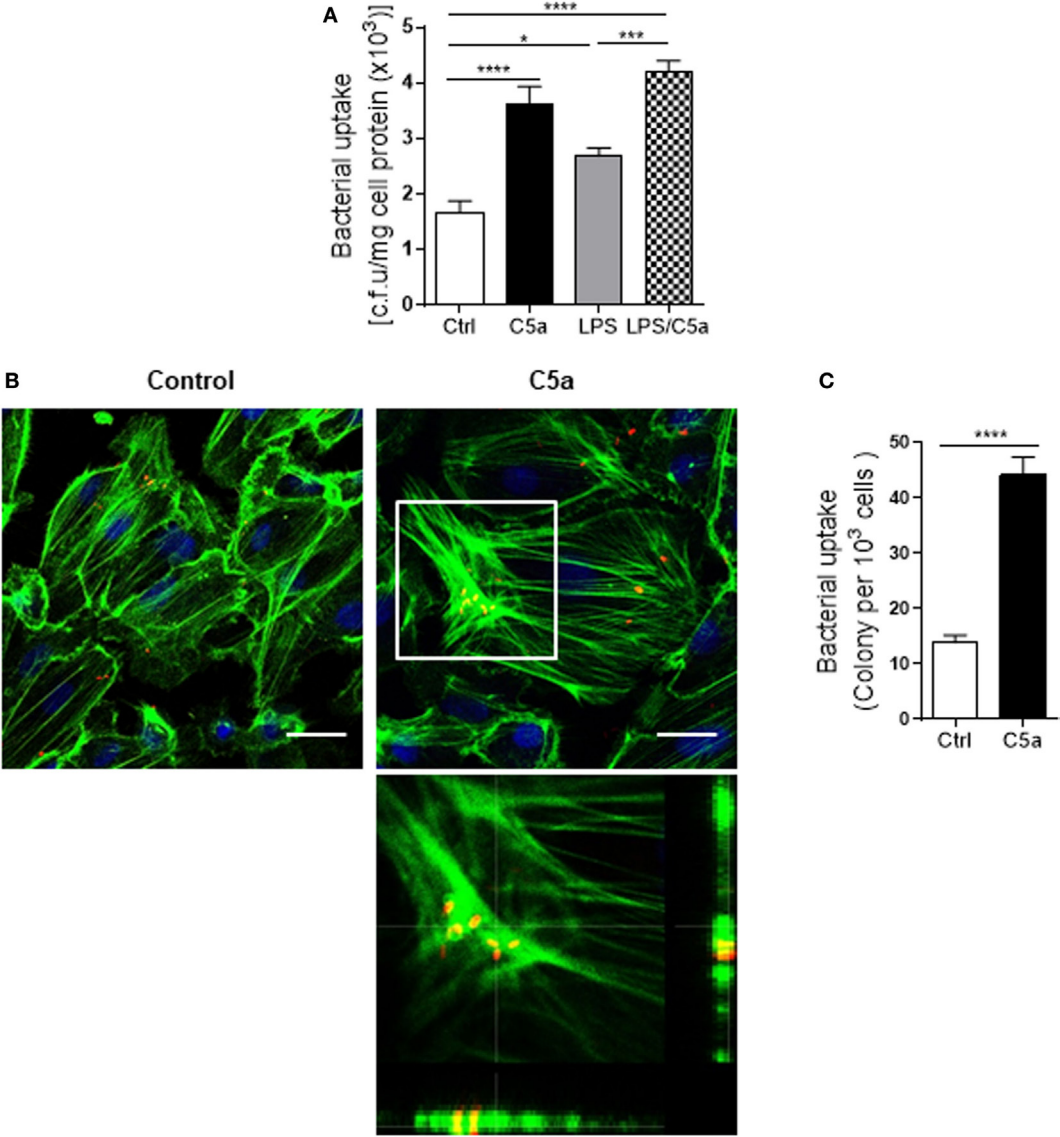
Effect of C5a stimulating renal tubular epithelial cell (RTEC) on bacterial internalization into the RTEC. **(A)** Bacterial uptake by RTEC, evaluated by bacterial plate count assay. RTEC were pre-treated with C5a (10 nM) and/or LPS (800 ng/mL) for 24 h and subjected to the assay. Data were analyzed by one-way ANOVA with Tukey’s multiple comparisons test (*n* = 8 individual wells) and representative of three independent experiments. **(B,C)** Bacterial uptake by RTEC, evaluated by fluorescence microscopy analysis. RTEC were pre-treated with or without C5a (10 nM) for 24 h and subjected to the assay. **(B)** Representative fluorescence images of (tri-iodothyronine and tetramethylrhodamine-labeled) bacteria internalization into RTEC. Bacteria (red), F-actin (green), and 4’,6-diamidino-2-phenylindole (blue) are shown. Scale bars, 25 µm. Lower panel image corresponding to the boxed region in the top-right image shows the cross-sectional views in z stack (bottom and side panel) of RTEC, F-actin, and bacteria, demonstrating J96 within the cytoplasm of RTEC. A representative of three experiments is shown. **(C)** Quantification of bacteria uptake by RTEC corresponding to the images in **(B)**. Results were expressed as number of bacteria per 10^3^ RTEC. Data were analyzed by unpaired two-tailed Student’s *t*-test (*n* = 15 individual images per group). *P < 0.05, ***P < 0.001, ****P < 0.0001.

**Figure 3:**
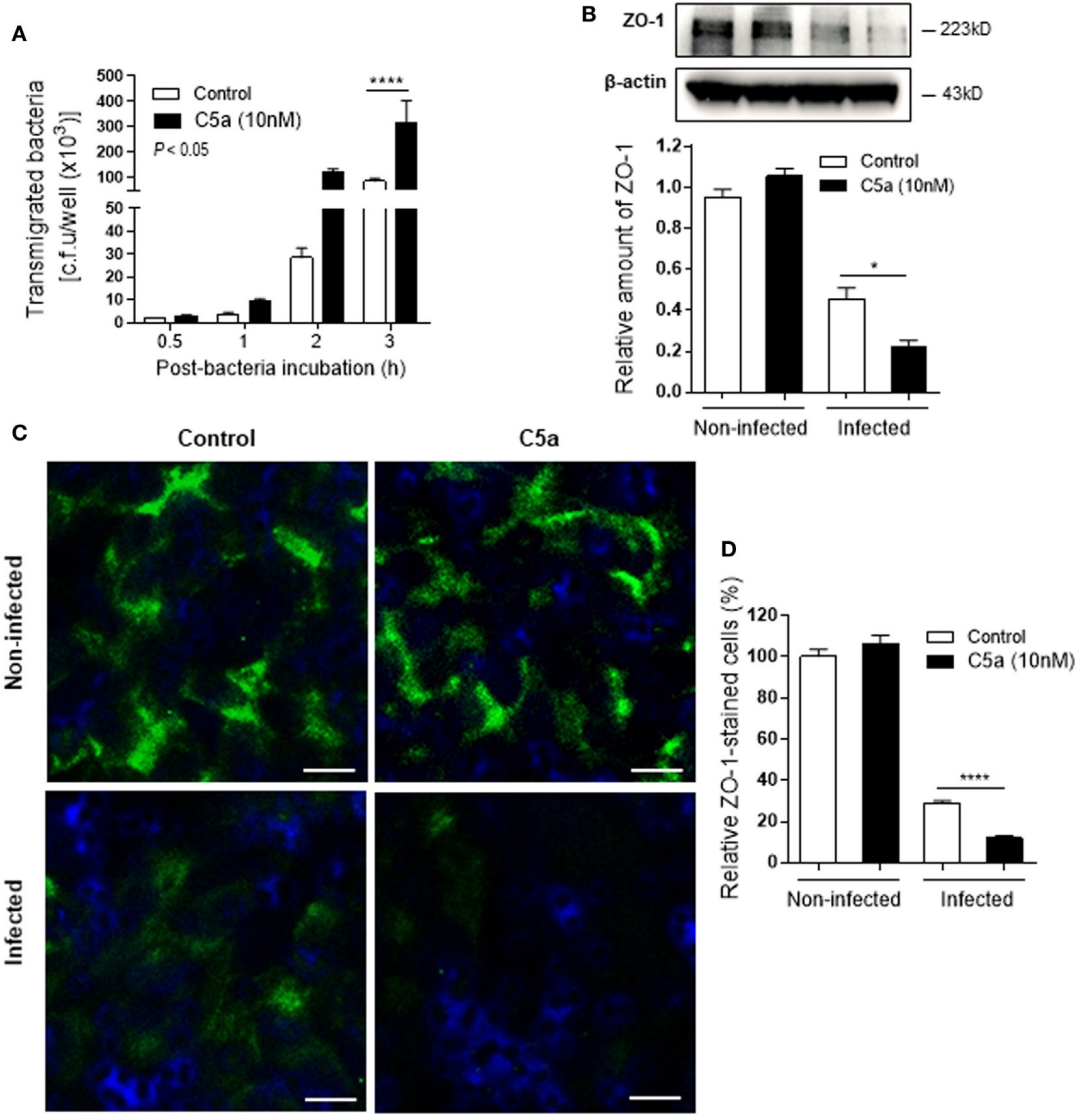
Effect of C5a stimulating renal tubular epithelial cell (RTEC) on bacterial transmigration through the monolayer of RTEC. Bacterial transmigration in RTEC that had been pre-treated with or without C5a (10 nM) for 24 h, then incubated with or without bacteria up to 3 h. **(A)** Transmigrated bacteria recovered from the lower chamber of RTEC cultures, evaluated by plate count assay. Data were analyzed by two-way ANOVA with multiple comparisons (*n* = 4 individual wells per group). **(B–D)** Epithelial barrier damage, evaluated at 2 h after incubation with bacteria by reduction of tight junction marker ZO-1 expression. **(B)** Western blot analysis for ZO-1 in RTEC. Data were analyzed by unpaired two-tailed Student’s *t*-test (*n* = 3 individual images per group). **(C)** Representative fluorescence images of ZO-1 (green) staining in RTEC. Scale bars, 10 µm. **(D)** Quantification of ZO-1-positive cells, shown as C5a treatment relative to control, corresponding to the RTEC shown in **(C)**. Data were analyzed by unpaired two-tailed Student’s *t*-test [*n* = 4 individual images (×200 magnification) per group]. *P < 0.05, ****P < 0.0001. All results shown are representative of three independent experiments.

Next we assessed the effects of C5a on bacterial adhesion and invasion in human RTEC by stimulation of the RTEC. RTEC were incubated with C5a, in the presence or absence of LPS, and then co-cultured with UPEC strain J96 and followed by a set of assays including bacterial binding, internalization, and transmigration. Bacterial binding to RTEC was assessed by plate count assay and fluorescence microscopy. C5a (alone) stimulation of RTEC significantly enhanced bacterial adhesion to RTEC in a dose-dependent manner (Figure 1A). Combined treatment with C5a (10 nM) and LPS (800 ng/mL) is more effective than treatment with LPS alone, the combined treatment also appears more effective than treatment with C5a alone, though there is no statistically significant difference (Figures 1B–D). Besides promoting adhesion, C5a stimulation of RTEC also significantly increased bacterial internalization into RTEC, regardless of in the presence or absence LPS, assessed by bacterial plate count assay and fluorescence microscopy (Figures 2A–C). Furthermore, C5a stimulation increased bacterial transmigration through the monolayer of RTEC (assessed by c.f.u. of transmigrated bacteria) and caused more severe epithelial barrier damage (assessed by ZO-1 expression) (Figures 3A–D).

Collectively, these data demonstrate C5a enhances UPEC adhesion to and invasion of human RTEC by stimulation of the RTEC.

### C5a-Mediated Upregulation of Man Expression in RTEC Contributes to the Enhancement of UPEC Adhesion to and Invasion of RTEC

**Figure 4:**
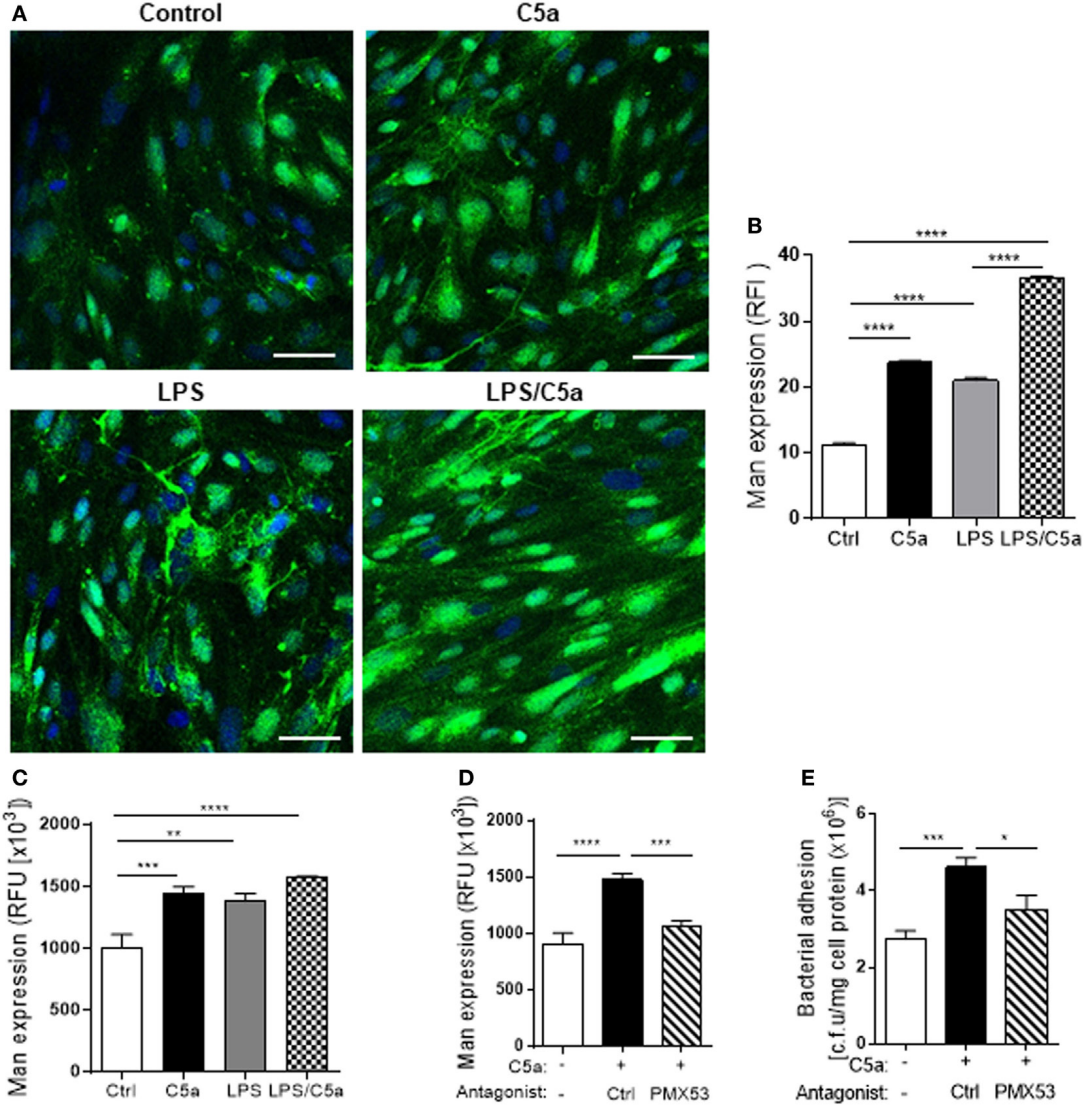
C5a stimulation upregulates Man expression in renal tubular epithelial cell (RTEC). RTEC were pre-treated with C5a (10 nM) and/or LPS (800 ng/mL) or with C5a, in the presence of C5aR1 antagonist (PMX53, 5 µM) or vehicle (Ctrl) for 24 h and subjected to assessment of Man expression and bacteria binding to RTEC. **(A,B)** Fluorescence microscopy analysis. **(A)** Representative fluorescence images of Man expression in RTEC (non-permeabilized). Man (green) detected by fluorescein-labeled *Galanthus nivalis* lectin (GNL) and 4’,6-diamidino-2-phenylindole (blue) are shown. Scale bars, 25 µm. **(B)** Quantification of Man expression, shown as relative fluorescence intensity in GNL-positively stained area corresponding to the images shown in **(A)**. **(C,D)** Fluorescence intensity-based microplate assay. **(C)** Man expression in RTEC that had been treated with C5a and/or LPS. **(D)** Man expression in RTEC that had been treated with C5a and PMX53. **(E)** Bacterial adhesion to RTEC, evaluated by bacterial plate count assay. **(B–D)** Data were analyzed by one-way ANOVA with Tukey’s multiple comparisons [**(B)** *n* = 6 coverslips per group, under ×100 magnification, **(C)** *n* = 9 individual wells per group, **(D,E)** *n* = 6–8 individual wells per group]. All results shown are representative of three independent experiments. *P < 0.05, **P < 0.01, ***P < 0.001, ****P < 0.0001. Abbreviations: Ctrl, control; RFI, relative fluorescence intensity; RFU, relative fluorescence units.

We next examined molecular mechanism by which C5a mediates the upregulation of bacterial adhesion/invasion in RTEC. Bacterial-host cell carbohydrate interactions are known to facilitate tissue invasion. Terminal α-Man are the carbohydrate ligand for type 1 fimbriae on UPEC (23, 24). Putative membrane proteins containing such ligands have been reported in the bladder in mouse and human (25). Our recent work has shown that terminal α-Man are also expressed in murine RTEC, the expression is upregulated by C5a stimulation. However, it is unknown whether this is true for human RTEC. We firstly confirmed the expression of Man in human RTEC by showing the cells were positively stained with fluorescence labeled GNL which specifically detects (α-1,3) Man (Figure S3 in Supplementary Material). We next assessed whether C5a influences the Man expression in human RTEC using two methods (i.e., fluorescence microscopy, fluorescence intensity-based microplate assay). Results obtained by both methods showed that C5a stimulation significantly increased Man expression in RTEC, the stimulatory effect was more profound in the presence of a small dose of LPS (800 ng/mL) (Figures 4A–C). Blockade of C5a/C5aR1 interactions with C5aR1 antagonist (PMX53), significantly reduced C5a-upregulated Man expression in RTEC and bacterial adhesion (Figures 4D,E), suggesting that C5a upregulates Man expression through interaction with C5aR1 in human RTEC.

**Figure 5:**
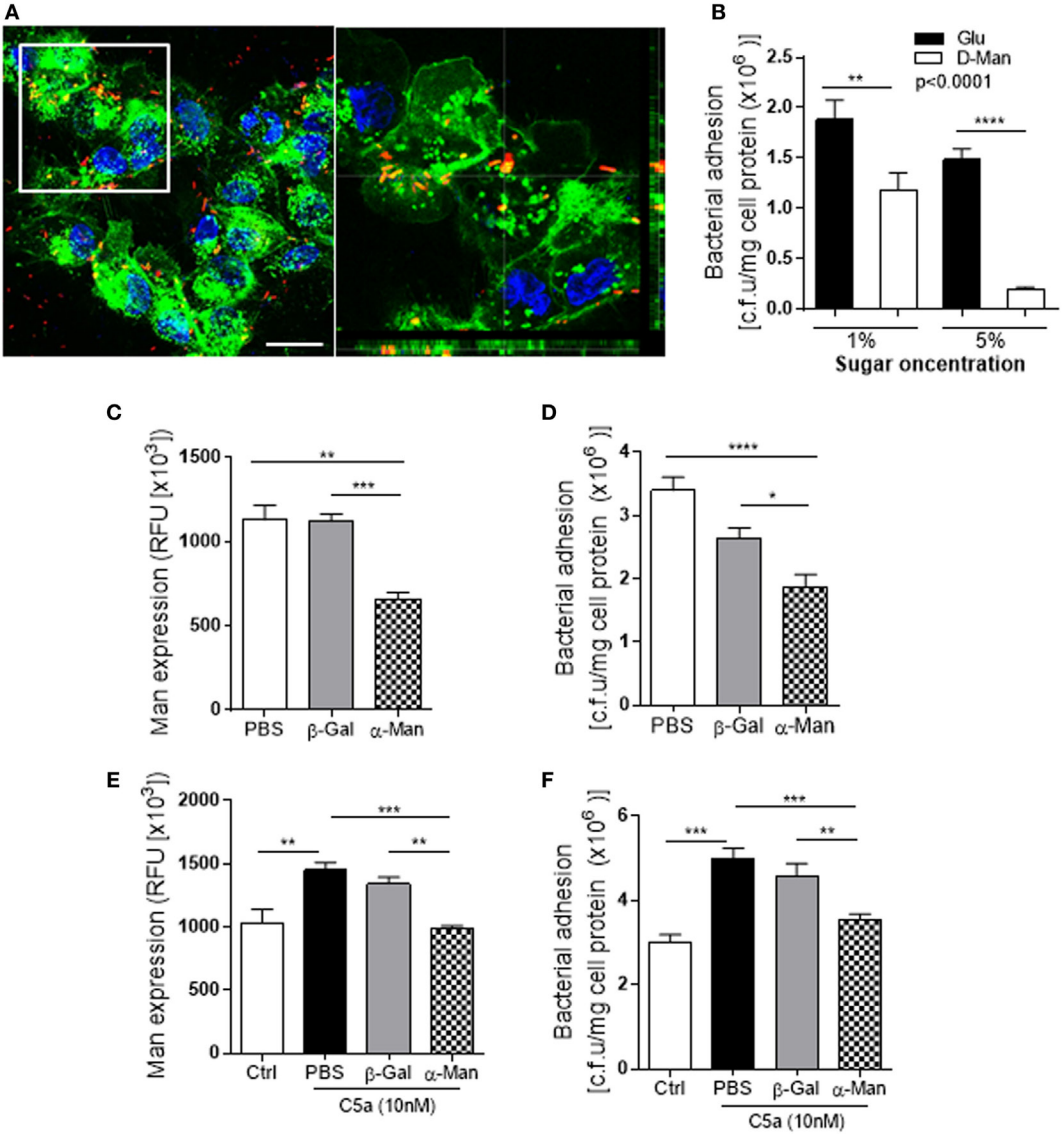
Uropathogenic *Escherichia coli* adhesion to renal tubular epithelial cell (RTEC) through binding to Man on the cell surface of the RTEC. **(A)** Confocal microscopic images of bound bacteria in RTEC (non-permeabilized) that had been incubated with labeled J96 for 1 h. Bacteria (red), Man (green) detected by fluorescein-labeled *Galanthus nivalis* lectin and 4’,6-diamidino-2-phenylindole (blue) are shown. Left image: compressed image. Scale bars, 10 µm. Right image: corresponding to the boxed region in the left image show the cross-sectional views in z stack (bottom and side panel) of RTEC, Man, and bacteria, demonstrating association of Man and J96 at cell surface of RTEC. **(B)** Bacteria binding to RTEC, evacuated by bacterial plate count assay. RTEC were incubated with d-mannose or glucose (1 or 5%) for 30 min before the addition of bacteria. Data were analyzed by two-way ANOVA with multiple comparisons (*n* = 8 individual wells per group). **(C,E)** Man expression in RTCE that had been pre-treated without C5a **(C)** or with C5a **(E)** for 24 h and then treated with buffer alone (PBS) or containing α-mannosidase (5 mM) or control enzyme (β-galactosidase) for 1 h, evacuated by fluorescence intensity-based microplate assay. Data were analyzed by one-way ANOVA with Tukey’s multiple comparisons (*n* = 6 individual wells per group). **(D,F)** Bacterial binding to RTEC that had undergone the same treatments as described in **(C,E)**, evacuated by bacterial plate count assay. **(D)** Without C5a pre-treatment. **(F)** With C5a pre-treatment. Data were analyzed by one-way ANOVA with Tukey’s multiple comparisons (*n* = 8 individual wells per group). *P < 0.05, **P < 0.01, ***P < 0.001, ****P < 0.0001. All results shown are representative of three independent experiments.

We also verified adhesion of UPEC to the RTEC *via* binding to Man on cell surface. The confocal Z-stack images showed a spatial relation between Man and J96 on RTEC cell surface (Figure 5A). Addition of d-mannose [which binds to lectin (Fim H of type 1 fimbriae)] in RTEC and UPEC co-culture led to a significant decrease in bacteria binding to RTEC (Figure 5B), demonstrating specific binding of bacteria to Man. Removal of surface Man from RTEC with α-mannosidase (which cleaves mannose containing glycoprotein and glycolipid) significantly reduced Man expression on RTEC and binding of J96 to RTEC (Figures 5C,D). Furthermore, removal of surface Man from RTEC inhibited the enhancement effect of C5a on Man expression and bacterial adhesion (Figures 5E,F), supporting the effect of C5a on bacterial adhesion is depended on upregulation of Man expression in RTEC.

Collectively, these data suggest that C5a/C5aR1 interaction-mediated upregulation of Man expression in RTEC contribute to the enhancement of bacterial adhesion/invasion in RTEC.

### Investigate Intracellular Signaling/Molecules Responsible for the Action of C5a on Man Expression in RTEC

**Figure 6:**
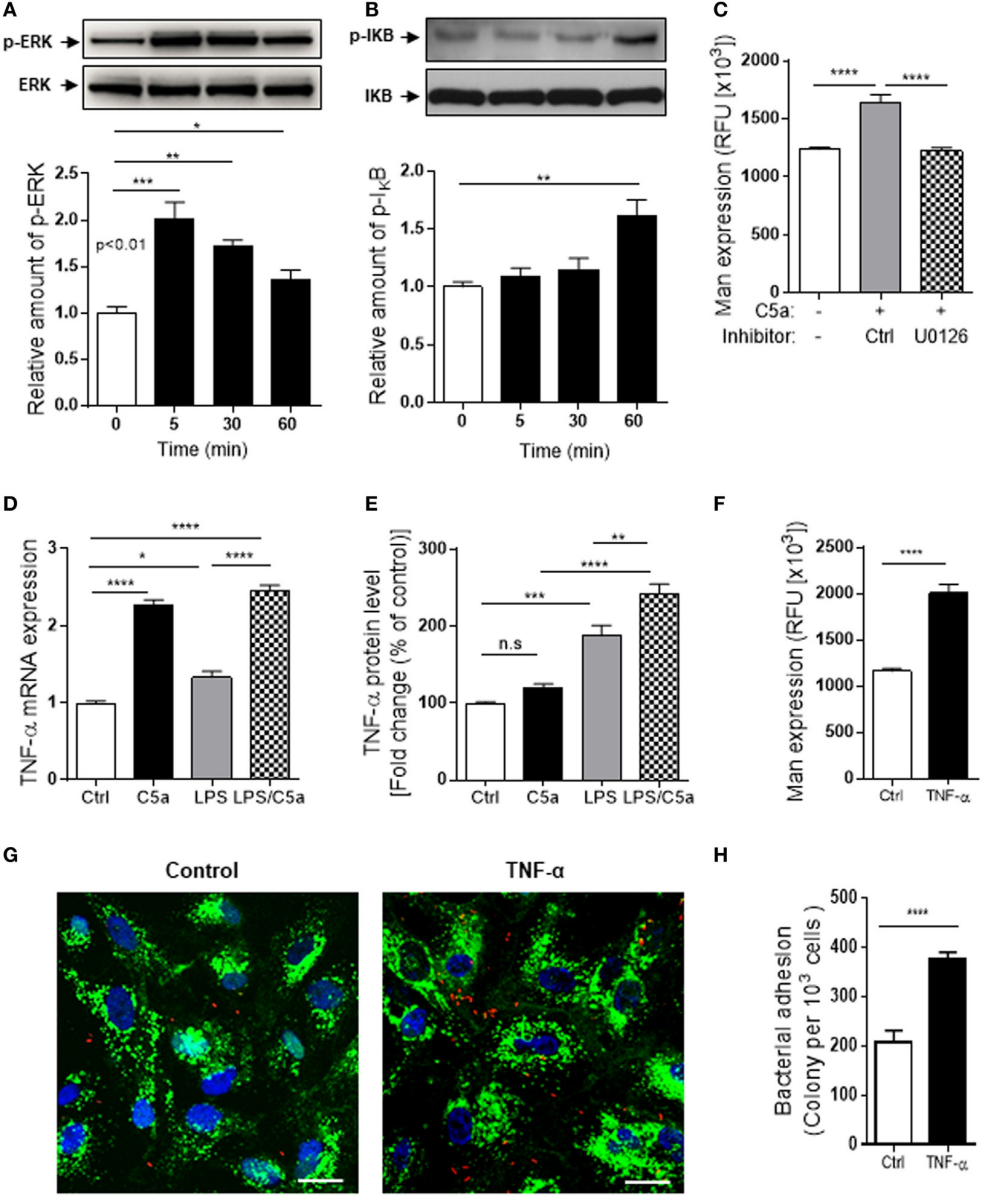
Intracellular signaling/molecules responsible for the action of C5a on Mannosyl residue expression. **(A,B)** Western blot analysis for ERK1/2 and IκB phosphorylation in renal tubular epithelial cell (RTEC) after C5a (10 nM) stimulation for up to 60 min. In each set of blots, the top row of bands corresponds to incubating membrane with appropriate anti-phospho-antibody and the bottom row of bands corresponds to incubating membrane with appropriate total antibody. Relative amounts of protein phosphorylation are shown in the lower panel of each set of blots. Data were analyzed by one-way ANOVA (*n* = 3/group, resulting from three independent experiments). **(C)** Effect of inhibition of ERK1/2 pathway on Man expression in RTECs assessed by fluorescence intensity-based microplate assay. RTEC monolayers were pre-incubated with C5a for 24 h in the presence of ERK1/2 pathway inhibitor (U0126, 10 μM) and the vehicle control (DMSO) then were used for quantification of Man expression. Data were analyzed by unpaired two-tailed Student’s *t*-test (*n* = 9–12 individual wells per group). A representative result of three experiments is shown. **(D,E)** Production of TNF-α in RTEC that had been treated with C5a (10 nM) and/or LPS (800 ng/mL) for 24 h and subjected to RT-qPCR **(D)** and ELISA **(E)**. Data were analyzed by one-way ANOVA with Tukey’s multiple comparisons (*n* = 3/group, resulting from three independent experiments). **(F)** Man expression in RTEC that had been pre-treated with recombinant human TNF-α (10 ng/mL) or vehicle control (BSA) for 24 h, evaluated by fluorescence intensity-based microplate assay. Data were analyzed by unpaired two-tailed Student’s *t*-test (*n* = 9 replicate wells/group). A representative result of three experiments is shown. **(G,H)** Bacterial adhesion to RTEC. **(G)** Representative microscopic images of bacteria adhesion to RTEC that had been pre-treated with TNF-α for 24 h, then incubated with tri-iodothyronine and tetramethylrhodamine-labeled J96 for 1 h. Bacteria (red), Man (green) detected by fluorescein-labeled *Galanthus nivalis* lectin, and 4’,6-diamidino-2-phenylindole (blue) are shown. Scale bars, 25 µm. **(H)** Quantification of bound bacteria corresponding to the images shown in **(G)**. Data were analyzed by unpaired two-tailed Student’s *t*-test [*n* = 6 individual images (×200 magnification) from two coverslips per group]. A representative result of three experiments is shown. *P < 0.05, **P < 0.005, ***P < 0.001, ****P < 0.0001.

Having demonstrated upregulation of Man expression in human RTEC by C5a/C5aR1 interactions, next we investigated which intracellular signaling pathways triggered by C5a could contribute to the Man expression in human RTEC. C5aR1 is a G-protein-coupled receptor for C5a, engagement of C5aR1 mediates various intracellular signaling events (e.g., PI3K, MAPKs, and NF-κB). We therefore examined phosphorylation of IκB [an indicator of NF-(κB activation), AKT (a downstream effector of PI3K), and ERK1/2 (a member of MAPKs)] in RTEC in response to C5a stimulation. Following C5a (10 nM) stimulation, the amount of p-ERK and p-IκB was increased and peaked at 5 and 60 min, respectively (Figures 6A,B). C5a stimulation had no effect on p-AKT (Figure S4 in Supplementary Material). We also assessed whether intracellular signaling of ERK1/2 responsible for the action of C5a on Man expression. Man expression in RTEC was significantly decreased by pre-treatment of the cells with specific inhibitor of ERK1/2 (U0126) (Figure 6C), indicating that intracellular signaling *per se* or through mediation of cellular inflammatory response could influence the Man expression in human RTEC. Further experiments were performed to examine cellular inflammatory responses to C5a stimulation in RTEC. The results showed that C5a stimulation significantly increased gene and protein expression of a set of proinflammatory mediators in the RTEC (Figure S5 in Supplementary Material), among those, the effect of C5a on TNF-α was most prominent (Figures 6D,E). Together, these results suggest the possibility that TNF-α driven by intracellular signaling mediated by C5a modulates Man expression in human RTEC. To explore the possibility, we assessed the effect of TNF-α on Man expression in RTEC. TNF-α stimulation significantly increased Man expression (Figure 6F) and accordingly bacterial adhesion (Figures 6G,H), demonstrating an important role of TNF-α in modulating Man expression and bacterial adhesion in human RTEC. Thus, our data support that C5a-mediated intracellular signaling (e.g., ERK1/2, NF-κB) modulates Man expression in human RTEC through mediating inflammatory mediators (e.g., TNF-α).

### Clinical Relevance of *In Vitro* Observations

**Figure 7:**
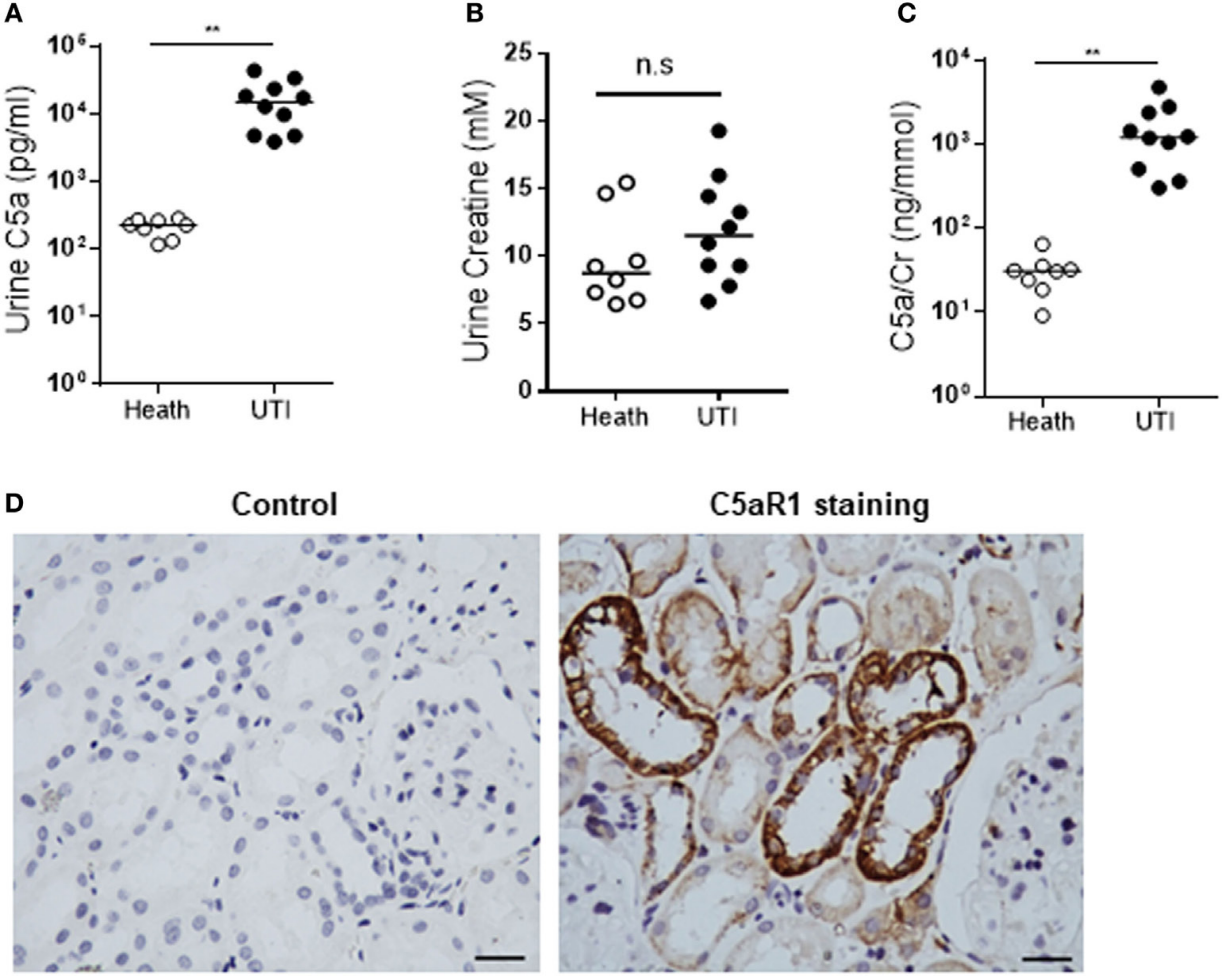
Detection of C5a and C5aR1 in human urinary tract. **(A–C)** C5a and creatinine concentrations in urine from urinary tract infection (UTI) patients and healthy controls. **(A)** Urinary C5a concentrations. **(B)** Urinary creatinine concentrations. **(C)** Ratios of urinary C5a/creatinine concentration. Data were analyzed by Mann–Whitney *U* two-tailed test. **P < 0.01, n.s. no significant. **(D)** C5aR1 expression in human kidney tissue determined by immunochemical staining. Control: negative control, tissue stained with non-specific mouse IgG2a. Scale bar, 50 µm.

**Figure 8:**
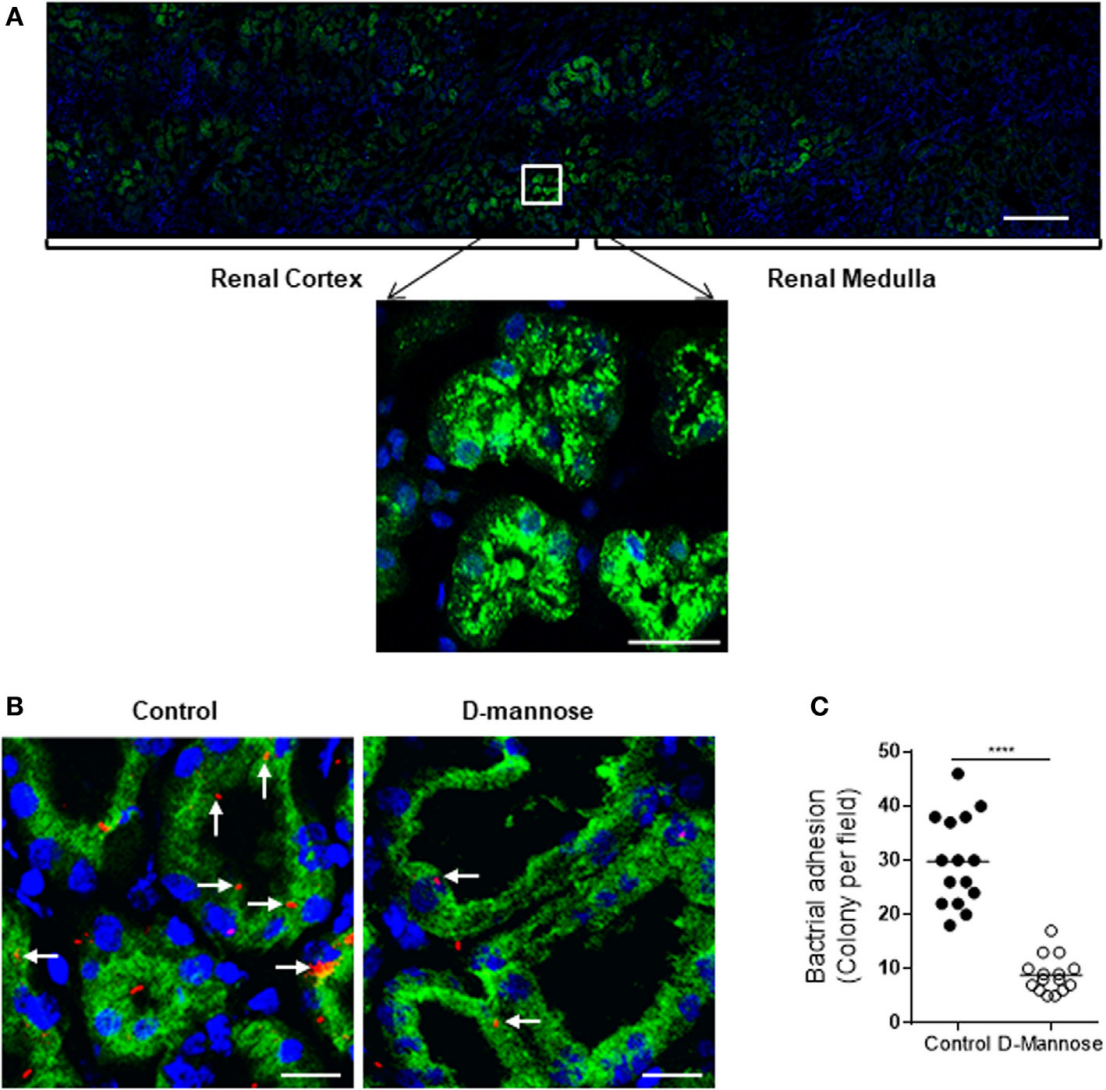
Man is required for type1 fimbriae-mediated *in situ* infection of human kidney tissues. **(A)** Fluorescence microscopy of normal kidney sections showing specific detection of Man (green) by fluorescein-labeled *Galanthus nivalis* lectin and counterstaining of nuclei with 4’,6-diamidino-2-phenylindole (blue). Man were mainly detected in the cortical and medullar tubules (top panel) and predominantly localized at the luminal surface of tubular epithelial cells (lower panel). Scale bars, 500 µm (top panel); 25 µm (lower panel). **(B,C)** Kidney sections were incubated with tri-iodothyronine and tetramethylrhodamine-labeled J96 (which were pre-treated with or without 5% d-mannose at 37°C for 30 min) for 1 h. Bacterial adhesion to tubular epithelium evaluated by fluorescence microscopy. **(B)** Represent images showing bacterial adhesion in the areas of cortical-medulla junction. Bacteria (red), Man (green), and nucleus (blue) are shown. Scale bars, 25 µm. **(C)** Quantification of bacterial adhesion to renal tissues corresponding to the images shown in **(B)**, results were expressed as number of colonies per field at ×200 magnification. Data were analyzed by unpaired two-tailed Student’s *t*-test (*n* = 15 viewing fields from three kidney sections per group). ****P < 0.0001.

To assess the *in vivo* relevance of our *in vitro* observations, we performed several *ex vivo* experiments using clinical samples. We examined the C5a levels in urine of 8 normal controls and 10 patients with active UTI (predominantly cystitis) by ELISA. C5a was detected in all urine samples including from the healthy controls and UTI patients. However, the urinary C5a levels were significantly higher in UTI patients than that in healthy controls (median concentration 228.5 vs. 15,230 pg/mL, controls vs. patients, *P* < 0.0001) (Figure 7A). Because all the samples are single spot urine samples, the detected C5a levels may not accurately reflect the C5a concentrations in the urine due to differences in urine flow rate over a day. Therefore, we examined the creatinine concentrations and determined the C5a/creatinine ratios in these urine samples, as urinary creatinine excretion in the presence of a table glomerular filtration rate is fairly constant in a given patient. Creatinine concentrations in the urine samples of UTI patients were compatible to that in healthy controls, but the C5a/creatinine ratios in the urine samples of UTI patients were significantly higher, compared with that in healthy controls (Figures 7B,C). Together, these results indicate there is increase in urinary C5a during UTI. In addition to detecting urinary C5a, we examined the expression of C5aR1 in (normal) human kidney biopsies. Immunohistochemistry showed that C5aR1 was clearly detected in the kidney tissue, mainly in renal tubules (Figure 7D). We also examined the Man expression in human kidney tissue and assessed the possibility that UPEC bind to renal tubular epithelium through Man by employing an *in vitro* model of kidney tissue infection. The human kidney was positively stained for GNL and positive signals were mainly found in the tubules of cortex and cortical medullary junction (Figure 8A). Fluorescence microscopy analysis showed that binding of UPEC to the tubular epithelium can be detected after 1 h infection (Figure 8B), blocking bacterial lectin by pre-incubation of UPEC with d-mannose markedly inhibited the binding of UPEC to tubular epithelium (Figure 8C), thus supporting the involvement of Man in UPEC adhesion/colonization in renal tubularepithelium.

## Discussion

Although it is well known that C5a/C5aR1 interactions mediate many cellular responses in different types of cells, its role in modulating surface carbohydrate expression of epithelial cells, thereby influencing bacterial adhesion/invasion is much less known. Our recent work in murine models of ascending UTI has suggested that C5aR1 signaling mediates upregulation of Man expression and subsequent bacteria adhesion contributes to the pathogenesis of renal infection (20, 21). In the present study, by using primary cultures of human RTEC and clinical samples (kidney biopsy and urine), we evaluated the relevance of observations on mice to human UTI. Our results provide supporting evidence for that human renal tubular epithelial C5aR1 signaling enhances UPEC adhesion to epithelial cells through upregulation of Man expression and its clinical implications.

As controversial observations have been reported on the expression of C5aR1 in human RTEC (26), we further examined the expression of C5aR1 in human kidney biopsies and primary cultures of RTEC in the present study. Our results of immunohistochemistry and immunocytochemistry are consistent with the reported positive expression of C5aR1 in kidney tissue and isolated tubular epithelial cells by most studies (27–31). Detection of C5aR1 expression in RTEC by RT-PCR (presented in this study) (Figure S2 in Supplementary Material) and in kidney tissues by *in situ* hybridization (reported previously) (31) further support for the expression of C5aR1 in human RTECs.

Consistent with observations in murine RTEC, in the present study we demonstrate that C5a/C5aR1 interactions upregulate Man expression and bacterial adhesion in human RTEC, suggesting the signaling/molecule mechanisms responsible for the action of C5a on Man expression and bacterial adhesion, namely C5a/C5aR1 interactions mediate the activation of ERK1/2/NF-κB signaling pathway and generation of proinflammatory cytokines, such as TNF-α, which upregulates Man expression and consequent bacterial adhesion. Although the issue of how TNF-α regulates surface Man was not addressed in our study, it has been shown that proinflammatory cytokines such as TNF-α can upregulate surface Man expression in endothelial cells through regulating α-mannosidase activity (32). The same would be expected in RTEC driven by C5aR1/ERK1/2/NF-κB signaling pathway.

Binding of bacteria to epithelial cells can mediate bacterial invasion of host cells through internalization by epithelial cells and/or transepithelial migration. Bacterial internalization by epithelial cells is increasingly recognized as an important feature of UTI. It can enhance bacterial survival by providing protection from host immune defenses and allow pathogens greater access to deeper tissues. Recent studies have suggested that intracellular *E. coli* can form a reservoir within the uroepithelium that may serve as a source for recurrent acute infections, a well-recognized clinical problem (4, 9). While epithelial barrier damage caused bacteria transmigration provides a route by which bacteria can access deeper structures and establish tissue-invasive infection. In the present study, we not only demonstrate the effect of C5a on bacterial adhesion, but also the effects of C5a on bacterial invasion, as evidenced by C5a stimulation caused an increase in bacteria within epithelial cells and transmigrated through epithelial cells. The observed increase of bacteria within epithelial cells is most likely to be the consequence of C5a/C5aR1 signaling-mediated upregulation of Man expression and bacterial binding. However, we cannot exclude the possibility of C5a/C5aR1 signaling-mediated enhancement of internalization through upregulating the expression of the receptors which have uptake functions, as we previously have shown that C3b-opsonized UPEC can interact with complement receptor (CD46) on the epithelial cell surface mediating UPEC internalization (8). In the case of bacteria transmigration, C5/C5aR1 signaling-mediated enhancement of Man expression and bacterial binding could lead to bacteria transmigration. In addition, C5a/C5aR1 signaling-mediated production of inflammatory mediators could cause epithelial barrier damage, thus facilitating bacteria transmigration.

We recognized the limitations of using primary cultured RTEC in our study. RTEC that we prepared from cortex of kidney biopsies are not pure proximal tubular epithelial cells (which are thought to be a primary target of UPEC), which may contain some other types of tubule cells, such as distal tubule cells, as distal tubules are also located in renal cortex. Therefore, the variations in cell responses among different RTEC preparations might be expected. However, renal cortex presents much more volumes of proximal tubules than distal tubules. In addition, we have tried to overcome the limitations by staining the cultured RTEC (permeabilized) with fluorescein-labeled Lotus Tetragonolobus Lectin (LTL) (a proximal tubular marker), only RTEC preparation that shows >75% of cells were positively stained with LTL were used for experiments.

*In vitro* findings in human RTEC promoted us to test the *in vivo* relevance. In the analyses of human kidney biopsies and urine, we made several observations, including: (i) urinary C5a levels are increased in UTI patients, (ii) C5aR1 is predominantly expressed in renal tubules, (iii) Man is readily detected in renal tubules across cortex and medullar in normal kidney biopsies, predominantly expressed in the luminal surface, (iv) UPEC binding to renal tubules in a mannose-dependent manner, which provide evidence supporting the possibility of C5a/C5aR1 interactions modulate Man-dependent UPEC adhesion *in vivo*.

In summary, our results demonstrate that human renal tubular epithelial C5aR1 signaling has a role in facilitating UPEC adhesion to and invasion of the epithelial cells through upregulation of Man expression and suggest that upregulation of Man is through C5aR1-mediated ERK1/2/NF-κB activation and release of proinflammatory cytokines, such as TNF-α. Our results from this study, together with our recent findings that C5aR1 has a pathogenic role in ascending UTI in mice (20, 21), suggest further studies to explore the therapeutic potential of targeting C5aR1 in UTI are warranted.

## Ethics Statement

This study was carried out in accordance with the recommendations of the guidelines of the Hospital Research Ethics Committee with written informed consent from all patients. Each patient gave an informed consent about the present study in accordance with the Declaration of Helsinki. The protocol was approved by the Hospital Research Ethics Committee at Xi’an Jiaotong University.

## Author Contributions

KL and WZ conceived and designed the study. YS, K-YW, Z-YD, Y-FG, and L-DZ performed experiments. YS and KL analyzed data. YS, MG, WZ, and KL prepared manuscript. L-DZ and TC provided tissue samples and contributed to interpretation of results.

## Conflict of Interest Statement

The authors declare that the research was conducted in the absence of any commercial or financial relationships that could be construed as a potential conflict of interest.
