# Supplementary material for: Epithelial C5aR1 Signaling Enhances Uropathogenic Escherichia coli Adhesion to Human Renal Tubular Epithelial Cells
Source: Front Immunol. 2018 May 1;9:949. doi: 10.3389/fimmu.2018.00949 (PMC5938350; doi:10.3389/fimmu.2018.00949)
Supplement: Supplementary file 1 [file presentation_1.PDF]

sFigure 1. Characterisation of primary cultures of human renal tubular epithelial cells

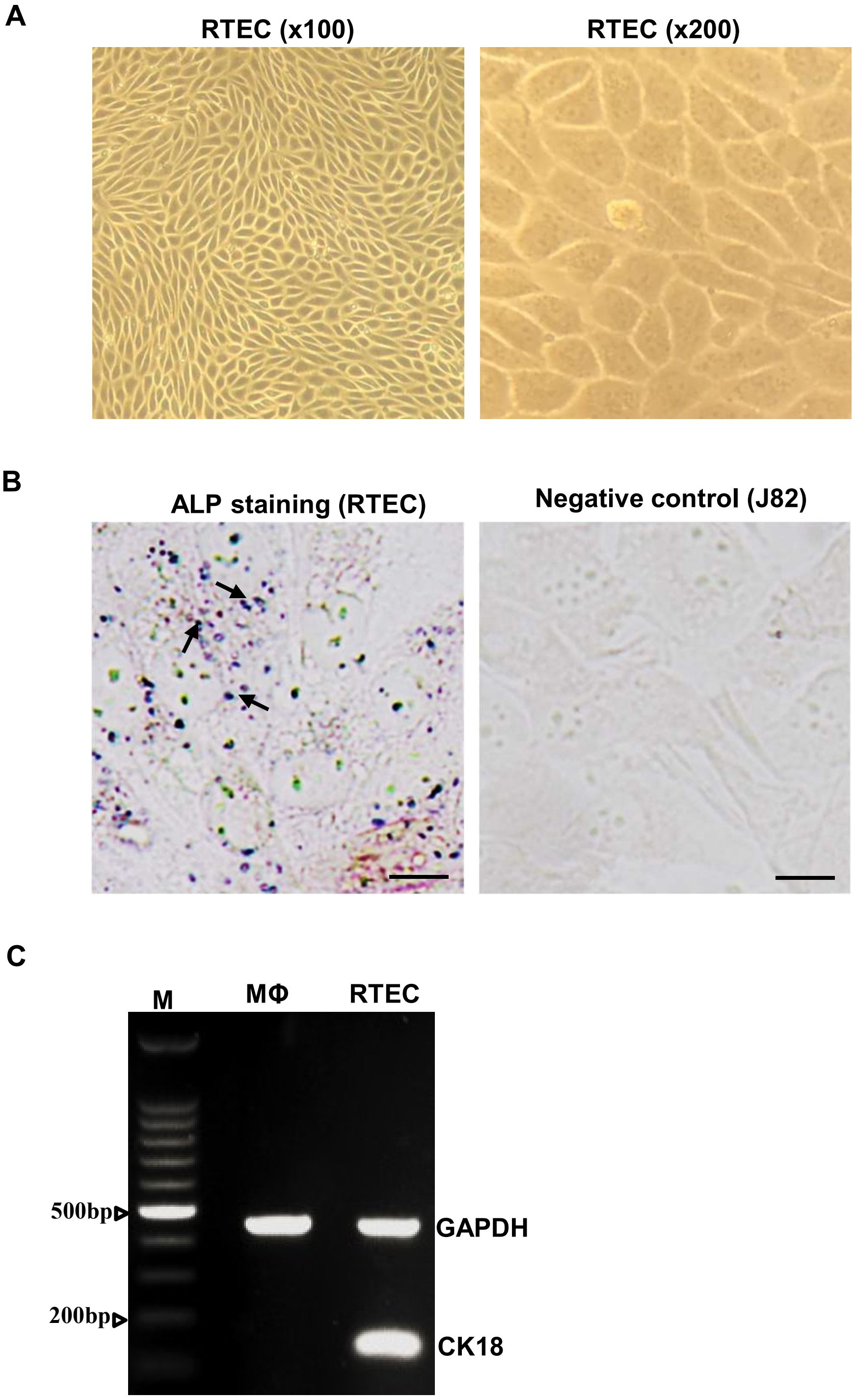

sFigure 1. Characterisation of primary cultures of human renal tubular epithelial cells
Human renal tubular epithelial cells (RTECs) were isolated from renal cortical and medulla junction tissues, fractionated then grown in culture medium selective for RTEC growth. RTECs from 3 sources exhibited identical features that did not alter up to passage 6. (**A**) Light microscopy of a 72-hour old primary culture (third passage), showing a confluent monolayer of cells exhibiting epithelial cobblestone morphology (original magnification ×100, left and x 200, right). (**B**) Representative images of alkaline phosphatase (ALP, an enzyme secreted by proximal tubular brush border) staining of RTECs, a human bladder epithelial cell line (J82) was included as the negative control. Scale bars, 50 µm. (**C**) Conventional RT-PCR was performed in primary cultured RTEC. The agarose gels showing the 134-bp cytokeratin-18 (CK18) band was present in RTEC cDNA, whereas it was absent from macrophage cDNA. GAPDH (453 bp) is included as an internal control, the 100 bp DNA markers (M) are shown alongside the gels. A representative of 3 independent experiments with separate cell preparations is shown.

sFigure 2. Detection of C5aR1 in primary cultures of human renal tubular epithelial cells

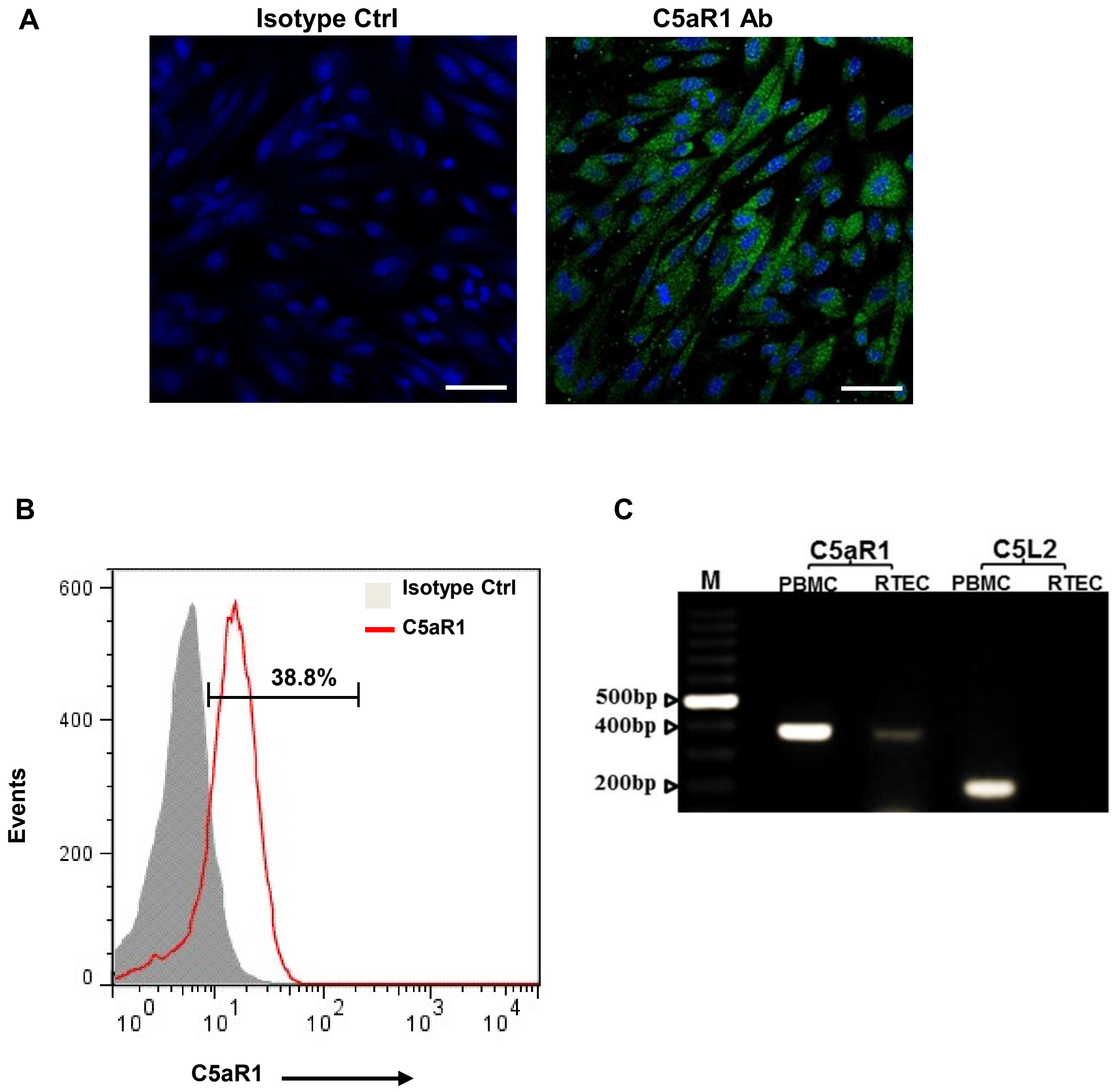

sFigure 2. Detection of C5aR1 in primary cultures of renal tubular epithelial cells
(**A**) Immunofluorescence microscopy images of C5aR1 staining using mouse anti-human C5aR1 antibody (P12/1, AbD Serotec) and FITC conjugated Donkey anti-mouse antibody. Mouse IgG2a was included as negative control. C5aR1 (green) and DAPI (blue). Scale bars, 50 μm. (**B**) Flow cytometry. C5aR1 was detected in peameblised RTECs by using PE-conjugated anti-human C5aR1 (S5/1, Biolegend), Mouse IgG2a was included as negative control. (**C**) RT-PCR. mRNA transcript for C5aR1 (401-bp) was detected in human PBMC and RTEC. However, mRNA transcript for C5R2 (200-bp) was only detected in PBMC but not in RTEC. A representative of 3 independent experiments with separate cell preparations is shown.

sFigure 3. Detection of mannosyl residues in renal tubular epithelial cells using fluorescein-labelled lectin GNL

A

GNL staining

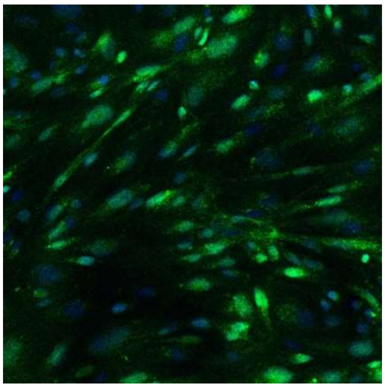

B

Negative control

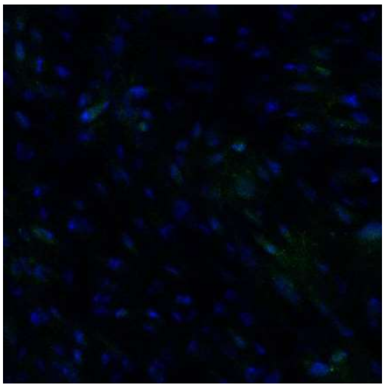

sFigure 3. Detection of mannosyl residues in renal tubular epithelial cells using fluorescein-labelled lectin GNL

Fluorescence microscopy of human RTECs primary cultured on coverslips after staining with fluorescein-conjugated lectin GNL. (**A**) Positive staining for mannosyl residues (MR) is seen on RTECs (green), with nuclei stained by DAPI (blue). (**B**) Similar coverslip but treated with GNL blocked by pre-incubation with 5% D-(+)-Mannose, confirming the specificity of GNL for detecting MR. Scale bars, 25μm.

sFigure 4. C5a stimulation has no remarkable effect on JNK and PI3K signalling in renal tubular epithelial cells

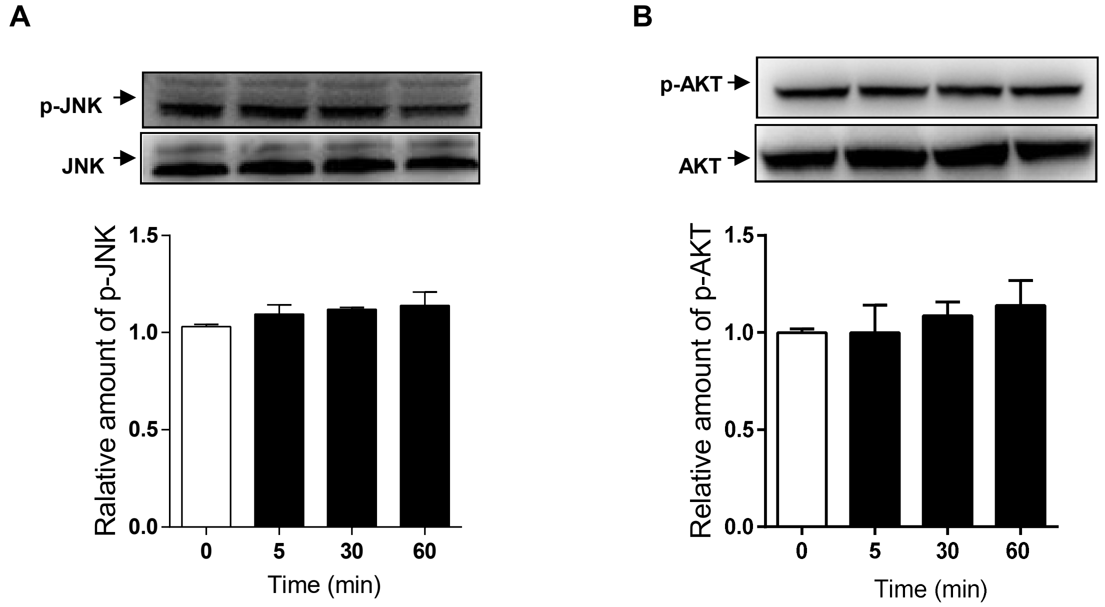

sFigure 4. C5a stimulation has no remarkable effect on JNK and PI3K signalling in renal tubular epithelial cells

Primary cultured human RTECs were stimulated with C5a (10 nM) up to 60 minutes. Western blot analysis for (**A**) SAPK/JNK [Thr183/Tyr185] and (**B**) AKT[Ser473] phosphorylation in RTEC. In each set of blots, the top row of the bands corresponds to incubating membrane with appropriate anti-phospho-antibody and the bottom row of the bands corresponds to incubating membrane with appropriate total antibody. Relative amounts of protein phosphorylation are shown in the lower panel of each set of blots. A representative of three independent experiments is shown.

sFigure 5. C5a/C5aR1 interaction up-regulates pro-inflammatory mediators production by renal tubular epithelial cells

A

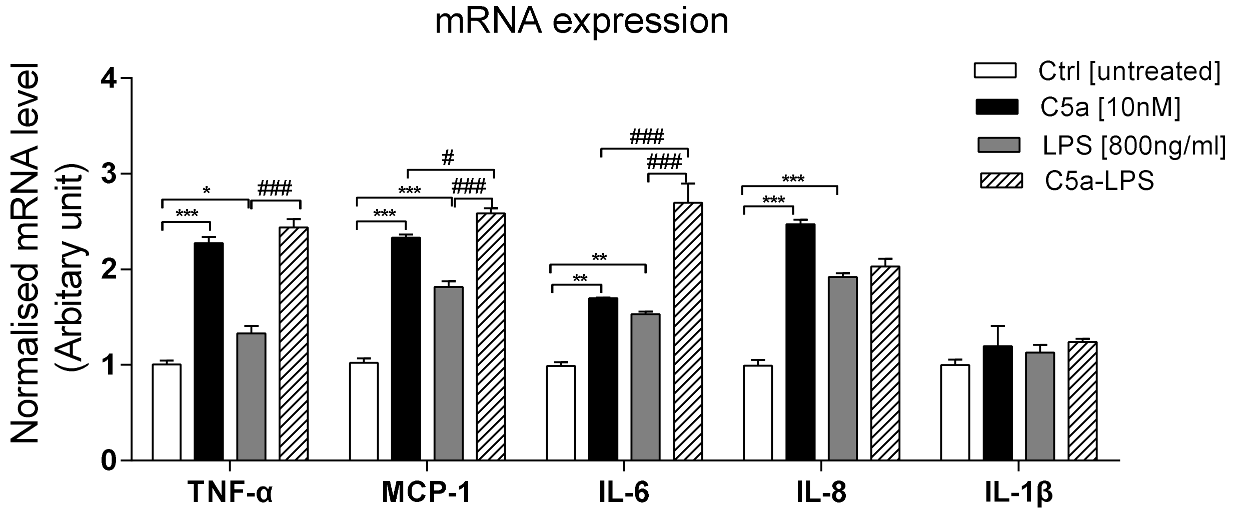

B

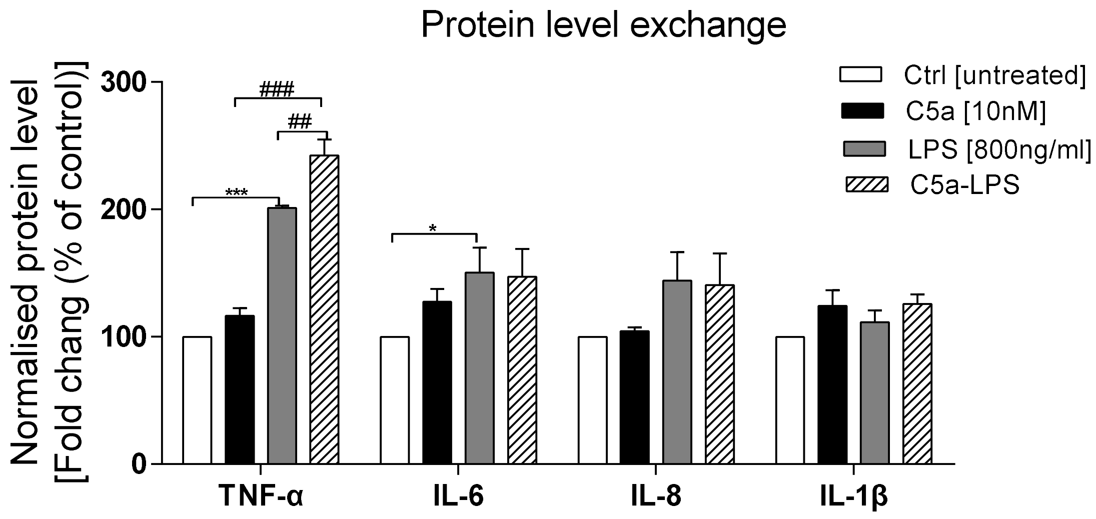

sFigure 5. C5a/C5aR1 interaction up-regulates pro-inflammatory mediators production by renal tubular epithelial cells

Primary cultured RTEC were incubated with C5a and LPS, alone and combined, for 24 hours and subjected to RT-PCR analysis, the culture supernatants were also collected to measure the pro-inflammatory cytokine production. (**A**) The relative changes in gene expression were analysed with the $2^{-\Delta\Delta\ CT}$ method. mRNA expression was expressed as fold change over the control (absence of C5a and LPS). (**B**) The protein levels of pro-inflammatory cytokine (e.g. TNF-α, IL-6, IL-8, IL-1β) was measured by BD™ cytometric bead array (CBA), and presented as fold change over the control. (**A,B**) Data were analysed by one-way ANOVA with Tukey's multiple comparisons test (A: n=3/group, B: n=5/group). *: indicates significant difference versus control (*P<0.05, **P<0.01, ***P<0.001). #: indicates significant difference versus C5a combined LPS treatment (# P<0.05, ## P<0.01, ### P<0.001). A representative of three independent experiments is shown.

Supplementary table 1. PCR primer sequences and product sizes

*Primer**	**Oligonucleotide Sequence** (5' ⟶ 3')	**Product Size (bp)**	**Gene bank code**
β-actin-1<br>b-actin-1	GGACTTCGAGCAAGAGATG<br>AGGAAGGAAGGCTGGAAGAG	138	NM_001101.4
TNF-α-1<br>TNF-α-2	CACAGTGAAGTGCTGGCAAC<br>AGGAAGGCCTAAGGTCCACT	185	NM_000594.3
IL-6-1<br>IL-6-2	TACCCCCAGGAGAAGATTCC<br>CCATCTTTGGAAGGTTCAGG	198	NM_000600.4
CXCL1-1<br>CXCL1-2	AGGGAATTCACCCCAAgAAC<br>TAACTATGGGGGATGCAGGA	132	NM_001511.3
IL-1β-1<br>IL-1β-2	CAGAAGTACCTGAGCTCGCC<br>AGATTCGTAGCTGGATGCCG	153	NM_000576.2
C5aR1-1<br>C5aR1-2	GAGCCCAGGAGACCAGAACATG<br>TACATGTTGAGCAGGATGAGGGA	441	NM_001736.3

* Primer-1 is identical to the coding strand; primer-2 is complementary to the coding strand.
